# The clinical effect of dexmedetomidine combined with parecoxib sodium on sedation, antianxiety and prevention of intubation stress in patients undergoing functional endoscopic sinus surgery: a randomised controlled trial

**DOI:** 10.1186/s12871-020-01080-0

**Published:** 2020-07-06

**Authors:** Xiaoxia Gu, Xiujuan Tan, Jinxian Chen, Jingjing Wang, Yue Lu, Liangqing Zhang

**Affiliations:** 1 Department of Anesthesiology, The First Affiliated Hospital, Jinan University, No. 601 West Huangpu Avenue, Tianhe District, Guangzhou City, 510632 Guangdong Province China; 2 Department of Anesthesiology, the Affiliated Hospital of Guangdong Medical University, No. 57 South People’s Avenue, Xiashan District, Zhanjiang City, 524001 Guangdong Province China

**Keywords:** Functional nasal endoscopy; dexmedetomidine; parecib sodium; sedation anxiety; intubation stress

## Abstract

**Background:**

To investigate the effect of intravenous injection of dexmedetomidine combined with parecoxib sodium on sedation and anxiety and stress response of tracheal intubation in patients undergoing functional endoscopic sinus surgery.

**Methods:**

One hundred twenty patients undergoing endoscopic sinus surgery were randomly divided into four groups: group DP, group D, group P and group N. The blood pressure (BP), heart rate (HR), blood oxygen saturation (SPO2), EEG, bispectral index (BIS), Ramsay sedation score and state anxiety questionnaire (SAI) were recorded before administration (T0), 10 min (T1), 20 min (T2) and 30 min (T3) after administration. After 30 min, endotracheal intubation was performed after anesthesia induction. The BP, HR, SPO2 were recorded 1 min before intubation (T4), intubation (T5), 3 min (T6) after intubation, 5 min (T7) after intubation, and blood samples were collected from patients before administration and after intubation 2 min to detect serum cortisol (Cor), adrenalin (E) norepinephrine (NE) and blood glucose (BS).

**Results:**

There was no significant difference in Ramsay sedation score before anesthesia, but the Ramsay sedation score in group D、DP was significantly higher than that in group P and group N, the BIS, BP, HR and anxiety scores were significantly lower than those in the group P and group N (*p* < 0.05). There was no significant difference in Ramsay sedation score, BIS value, anxiety score and BP, HR between group D and group DP (*p* > 0.05). Compared with T4, there was no significant difference in BIS and BP, HR in group D, group DP and group P (*p* > 0.05), but the BIS, BP and HR in group N were significantly higher than T4, (*p* < 0.05). Three minutes after intubation there was no statistical difference in the changes of Cor, E, NE and BS values compared with before intubation in group P and group DP (*p* > 0.05), but the changes of Cor, E, NE and BS values were significantly lower than that in group N (*p* < 0.05). Compared with T0, the values of NE, E, Cor, BS decreased in group D, DP and P at T4, group DP decreased more significantly than group D (*p* < 0.05). while the NE, E, Cor, BS of T6 are at the same level as the base value. In group N, the NE, E, Cor, BS of T4 were at the same level of T0, but significantly higher at T6.And at T6, NE and E in group D, P and N were significantly different from those in group DP (*p* < 0.05).

**Conclusion:**

Preoperative intravenous infusion of dexmedetomidine combined with parecoxib sodium by functional nasal endoscopy can not only calm and resist anxiety, but also better prevent stress response of endotracheal intubation, which is a safe and effective way of preoperative medication.

**Trial registration:**

ChiCTR-OPN-17010444. Prospectively registered on 16 January 2017.

## Background

Functional endoscopic sinus surgery (FESS) is a new technology rising in the field of nasal surgery, which has become the mainstream technology in the treatment of sinusitis. Because of the abundant blood vessels in the pathological part of the nasal cavity, it is easy to bleed. Therefore, in order to ensure the smooth operation of FESS, it is required that patients should be quiet and have less bleeding to keep the operation field clear. At the same time, keep the respiratory tract unobstructed to prevent the obstruction and aspiration of the respiratory tract [1]. Since the Department of Anesthesiology began to use DEX and parecoxib sodium in our hospital, it was found that the combination of the two drugs was worth further discussion in clinical practice. Therefore, we used dexmedetomidine combined with parecoxib sodium in patients undergoing FESS surgery. From the aspects of preoperative sedation, antianxiety and intubation stress, we evaluated the clinical effect of dexmedetomidine combined with parecoxib sodium in FESS surgery, in order to find a suitable preoperative anesthesia medication for FESS. It could provides a direct clinical basis and reference for anesthesiologists to implement comfortable and safe anesthesia.

## Methods

### General information

The trial adheres to CONSORT guidelines and was approved by the Ethics Committee of the Affiliated Hospital of Guangdong Medical University. From June 2017 to December 2019, 120 patients (aged 18–60, ASA I-II) were selected for FESS operation in otolaryngology department of our hospital. All patients participating in the trial were signed the informed consent before operation. The trial was registered prior to patient enrollment at clinicaltrials.gov (Effects of dexmedetomidine combined with parecoxib sodium in patients undergoing endoscopic nasal surgery rapid rehabilitation and prognosis, Principal investigator: GU Xiao-Xia, Date of registration:16 January 2017). Trial’s clinical trial registration number is ChiCTR-OPN-17010444.The patients were randomly divided into four groups, Dexmedetomidine + parecoxib sodium group (group DP), dexmedetomidine group (group D), parecoxib sodium group (group P) and normal saline group (group N)(*n* = 30). Randomization was carried out by a computer-generated list of random numbers which were sealed in an envelope. Before anesthesia, an anesthesiologist who was blinded to the study opened the sealed envelope and performed the anesthesia.

Inclusion criteria: ① age 18–60, gender unlimited, weight 45-75 kg, body mass index (BMI) 18–25 Kg / m^2^; ② ASA I-II; ③ tracheal intubation and intravenous general anesthesia for FESS operation, the operation time is 1-3 h, and the tracheal tube must be removed after the operation; ④ non emergency patients prepared before the routine operation; ⑤ clear mind, active cooperation; ⑥ no abnormality of heart, lung, liver and kidney function before the operation, no hypertension, anemia, endocrine disease and coagulation function; ⑦ voluntary Participated in and signed the informed consent form.

Exclusion criteria: ① Those who are known to be allergic to experimental drugs and other drugs during operation; ② those who have serious cardiovascular and respiratory diseases; ③ those whose liver and kidney functions are seriously damaged or whose water, electrolyte and acid-base balance are disordered; ④ those who have serious mental diseases or have language communication disorders, who can not understand the sedation score and VAS score; ⑤ those who often take analgesics or sedatives; ⑥ those who have bradycardia Atrioventricular block; ⑦ non steroidal anti-inflammatory drug allergy; ⑧ under primary school culture, unable to complete the questionnaire independently; ⑧ pregnant or lactating women; ⑨ researchers think it is not suitable to participate in this experiment.

### Anesthesia method

The patients were randomly divided into four groups: group DP, group D, group P and group N. All patients were forbidden to drink for 2 h and fasted for 8 h before operation without any preoperative medication. All patients were sent to the anesthesia preoperative room without any premedication 30 min before the surgery. Standard monitoring consisted of five-lead electrocardiography (ECG), oxygen saturation (SpO2), noninvasive blood pressure (BP) and heart rate (HR). Bispectral index (BIS) and Ramsay Sedation score were also used. First, One anesthesiologist who was unaware of the clinical nature of the study monitored and conducted the case. Study drug diluted with 0.9% normal saline to the same volume, Patients did not access the operating room until surgery. One anesthesia nurse who was not involved in the experiment prepared all the study drugs. The anesthesiologist in the operating room who was blinded to group assignment collected the date.① group DP: 30 min before anesthesia induction, DEX 1 μ g / kg (real weight) (Jiangsu Hengrui Pharmaceutical Co., Ltd., Guoyao Zhunzi h20090248, product specification: 2 ml: 200μg) was injected intravenously within 15 min, and then 40 mg of parecoxib sodium (Pfizer Pharmaceutical Co, Ltd. specification: 40 mg dosage form: injection approval No.: Bh20080116) (diluted to 2 ml with saline) was injected ② group D: 30 min before anesthesia induction, DEX 1 μ g / kg was injected intravenously and 2 ml saline was injected intravenously within 15 min ③ group P: 30 min before anesthesia induction, the same amount of saline was pumped intravenously, and then 40 mg of parecoxib sodium (diluted to 2 ml) was injected intravenously within 15 min. ④group N: 30 min before anesthesia induction, the same amount of saline was pumped intravenously, and then 2 ml of saline was injected intravenously within 15 min.30 min later, the operator gave anesthesia induction: sufentanil 0.5 μg / kg intravenously, etomidate 0.3 mg / kg intravenously 2 min later, cisatracurium 0.15 mg / kg after the disappearance of consciousness, and tracheal intubation 3 min later. The patients’ BP, HR and SpO2 were recorded at 1 min before intubation (T4), intubation (T5), 3 min after intubation (T6) and 5 min after intubation (T7). The blood samples of patients were collected before administration (T0), 1 min before intubation induction (T4) and 3 min after intubation (T6) to detect serum cortisol (COR), adrenaline (E) and noradrenaline (NE) And blood glucose (BS).

### Observation indicators

Another anesthesiologist (observer) observed the sedation scores of SBP, DBP, HR, SpO2, BIS and Ramsay before administration (T0), 10 min (T1), 20 min (T2), and 30 min (T3). State Anxiety Inventory (SAI) was used to assess the severity of anxiety symptoms 15 min before administration. The patients were asked to fill in SAI again 30 min after administration. The occurrence and management of adverse reactions before operation were recorded in real time, including respiratory depression, hypooxia, hypotension and arrhythmia. Sedation and anxiety assessment tool: State Anxiety Inventory (SAI) was used to assess the severity of current anxiety symptoms. The State Trait Anxiety Inventory (STAI), compiled by Charles Spielberger et al. In 1977 and revised in 1983, is characterized by its simplicity, high validity, easy analysis, fairly intuitive reflection of the subjective feelings of anxiety patients, especially the ability to integrate the current state anxiety inventory (SAI). It is a relatively reliable indicator to distinguish from the consistent trait anxiety inventory (TAI) [2]. SAI describes an unpleasant short-term emotional experience, such as tension, fear, anxiety etc., which is often accompanied by hyperactivity of the autonomic nervous system, generally transient, and can be used to evaluate the level of anxiety under stress. TAI is used to describe relatively stable, as a personality characteristic with individual differences of anxiety tendency, used to assess people’s frequent emotional experience. There are 20 items in the SAI questionnaire. The whole questionnaire is graded at grade 1–4 (state anxiety: 1-none, 2-some, 3-moderate, 4-very obvious; trait anxiety: 1-almost none, 2-some, 3-often, 4-almost always). The subjects choose the most appropriate grade according to their own experience. Ten positive and 10 negative emotion questionnaires. All positive items are scored in reverse order. The cumulative score of the state anxiety table was calculated. The minimum score was 20, and the maximum score was 80. The higher the score, the higher the anxiety level of the subjects.

### Statistical methods

Our primary outcome measures were stress response of tracheal intubation; the secondary outcomes were sedation and anxiety levels. The sample size estimation was based according to norepinephrine, the most intuitionistic one of the stress responses.

Our preliminary study found that norepinephrine levels in 10 patients undergoing FESS were 446.34 ± 13.25. A reduction of 10% in norepinephrine (401.71 ± 11.92) after preoperative intravenous infusion of DEX combined with parecoxib sodium in the treatment group was considered clinically significant, and this required a sample size of 29 pergroup to achieve a power of 80% and a type I error of 5%. To compensate for the possibility of dropout, we recruited 120 patients, 30 patients per group. We use SPSS22.0 statistical software analysis our data. The demographic characteristics data were evaluated using unpaired *t*-test for between-group and paired *t*-test for within group comparisons. The 2 test was used to analyze categorical variables. Student’s test and ANOVA were used for unpaired quantitative variables. Data are presented as Mean ± SD (SEM), count (%), or as Median (IQR (range)). *p* < 0.05 was statistically significant.

## Results

### Comparison of general data

There was no significant difference in age, height, weight, ASA grade among the four groups (*p* > 0.05). See Table 1.

**Table 1:** Comparison of general conditions of the four groups ($\overline{x}\pm s$)

Group	Cases	Age (years)	Height (cm)	Weight (kg)	ASA (I / II)
D	**30**	38.8 ± 8.9	155 ± 5.0	53.7 ± 7.4	20/10
DP	**30**	37.5 ± 7.6	156 ± 3.3	53.5 ± 6.9	21/9
P	**30**	38.3 ± 8.3	157 ± 3.8	54.9 ± 7.6	19/11
N	**30**	37.9 ± 6.7	156 ± 4.3	54.3 ± 5.9	20/10

Compared with group N, *p* > 0.05

### Sedation after administration in four groups

Ramsay Sedation score was maintained at 2–4 points and BIS value was maintained at about 70–80 in group D and DP, while Ramsay Sedation score was maintained at 1–2 points and BIS value was greater than 89 in P and N groups. Compared with group N, there were significant differences in Ramsay Sedation score and BIS value of group DP at T1 time point (*p* < 0.05); there were significant differences in Ramsay Sedation score and BIS value of group D and DP at T2 and T3 time point (*p* < 0.05); There were no significant difference in Ramsay Sedation score and BIS between group P and N (*p* > 0.05). See Table 2 for details.

**Table 2:** Changes of sedation indexes in the four groups ($\overline{x}\pm s$)

Group	Cases	Items	T0	T1	T2	T3
D	30	Ramsay	1.21 ± 0.41	1.45 ± 0.30	2.11 ± 0.55^ab^	2.90 ± 0.42^ab^
		BIS	93.9 ± 3.3	89.8 ± 2.5^b^	81.5 ± 3.3^ab^	78.3 ± 5.6^ab^
DP	30	Ramsay	1.25 ± 0.36	1.52 ± 0.51^b^	2.05 ± 0.60^ab^	2.81 ± 0.37^ab^
		BIS	92.6 ± 2.5	88.0 ± 2.7^b^	80.3 ± 4.0^ab^	75.4 ± 4.8^ab^
P	30	Ramsay	1.23 ± 0.44	1.45 ± 0.49	1.48 ± 0.50	1.55 ± 0.48
		BIS	93.2 ± 3.5	91.6 ± 3.1	89.4 ± 3.3	89.1 ± 4.3
N	30	Ramsay	1.19 ± 0.39	1.55 ± 0.49	1.41 ± 0.50	1.45 ± 0.48
		BIS	92.5 ± 3.6	92.6 ± 3.1	89.9 ± 3.5	89.5 ± 2.3

Compared with group N, ^a^*p* < 0.05, compared with T0, ^b^*p* < 0.05

### Anxiety scores of the four groups after administration are shown in Table 3

Compared with group N, the anxiety scores of patients in group D and group DP decreased significantly (*p* < 0.05), especially those in group DP, but there was no significant difference (*p* > 0.05) between the two groups. There was no significant difference in SAI score before and after treatment in group P (*P* > 0.05). See Table 3 for details.

**Table 3:** Changes in anxiety scores of the four groups before and after administration ($\overline{x}\pm s$)

Group	Cases	SAI Before administration after administration		VAS Before administration after administration	
D	30	45.8 ± 3.8	31.2 ± 3.9^ab^	44.3 ± 7.1	28.1 ± 5.1^ab^
DP	30	44.7 ± 2.9	28.4 ± 4.2^ab^	45.6 ± 5.3	26.8 ± 7.1^ab^
P	30	45.2 ± 3.6	43.1 ± 4.9	45.2 ± 5.9	40.4 ± 7.8
N	30	44.7 ± 3.3	43.0 ± 2.2	45.8 ± 4.5	40.1 ± 6.3

^a^*p* < 0.05 was compared with group N, and ^b^*p* < 0.05 was compared with that before administration

### The influence of four groups of patients on hemodynamics after administration is shown in Table 4

Compared with before intubation (T0), the SBP, DBP and HR of group N were significantly higher at 3 min (T6) and 5 min (T7) after intubation ((*p* < 0.05). The SBP, DBP of group D and group N were significantly higher at the time of intubation (T5) and 3 min (T6) after intubation than before intubation (T0)(*p* < 0.05), While the SBP and DBP of DP and P were significantly higher than those of group N (*p* < 0.05), but kept the same level as T0 of the same group. HR at intubation and after intubation in group D and DP was significantly lower than that in the same group (T0), with statistical significance (*p* < 0.05); HR in group P had no significant change at each time point, with no statistical significance (*p* > 0.05); compared with T0, HR in group N were significantly faster at intubation (T5) and after intubation (T6 and T7) with statistical significance (*p* < 0.05). See Table 4 for details.

**Table 4:** Hemodynamic changes before and after administration in the four groups ($\overline{x}\pm s$)

Group	Items (mmHg/bpm)	T0	T1	T2	T3	T4	T5	T6	T7
D	SBP	125.3 ± 10.1	122.7 ± 9.8	119.1 ± 7.5	119.6 ± 10.3	135.9 ± 11.5^b^	133.6 ± 11.3^b^	124.7 ± 10.5^a^	122.4 ± 10.7^a^
	DBP	75.9 ± 8.5	75.6 ± 10.5	74.2 ± 7.4	72.7 ± 8.7	82.2 ± 9.7^b^	82.7 ± 8.7^b^	72.7 ± 8.7^a^	72.7 ± 8.7^a^
	HR	85.6 ± 13.3	79.1 ± 13.9	75.1 ± 9.7	75.5 ± 8.7	81.1 ± 6.7^a^	82.5 ± 8.8^a^	81.4 ± 8.1^a^	80.7 ± 7.7^a^
DP	SBP	124.5 ± 12.5	122.5 ± 11.2	122.7 ± 9.6	121.1 ± 10.8	121.2 ± 11.3^a^	122.7 ± 10.2^a^	121.5 ± 11.8^a^	120. ± 11.1^a^
	DBP	78.6 ± 9.2	76.0 ± 8.7	73.5 ± 7.9	74.9 ± 7.3	75.7 ± 8.4^a^	76.1 ± 5.3^a^	76.6 ± 2.9^a^	76.3 ± 4.3^a^
	HR	87.0 ± 9.7	83.5 ± 10.2	83.4 ± 11.2	82.7 ± 11.4	83.4 ± 10.3^a^	84.5 ± 10.4^a^	83.7 ± 11.5^a^	84.1 ± 10.2^a^
P	SBP	123.6 ± 9.8	120.3 ± 9.2	120.2 ± 10.4	119.3 ± 10.6	122.6 ± 10.1^a^	124.8 ± 10.5^a^	125.3 ± 11.4^a^	121.9 ± 10.1^a^
	DBP	79.4 ± 7.1	76.6 ± 6.4	75.4 ± 6.5	76.1 ± 8.7	79.6 ± 8.2^a^	80.7 ± 8.7^a^	79.4 ± 5.7^a^	79.8 ± 8.2^a^
	HR	87.3 ± 10.6	86.5 ± 13.3	84.5 ± 10.6	85.3 ± 10.4	89.8 ± 10.3	90.3 ± 9.4	89.3 ± 1.8	89.6 ± 7.5
N	SBP	124.6 ± 8.8	123.1 ± 9.8	125.2 ± 10.4	123.3 ± 10.9	141.7 ± 10.3^b^	143.6 ± 11.9^b^	139.9 ± 12.1^b^	138.7 ± 10.4^b^
	DBP	77.4 ± 8.1	78.6 ± 11.0	78.4 ± 6.5	76.5 ± 5.7	85.5 ± 7.3^b^	86.5 ± 5.7^b^	85.5 ± 5.9^b^	83.5 ± 4.8^b^
	HR	86.3 ± 10.1	85.1 ± 13.9	86.5 ± 10.6	84.9 ± 9.9	95.5 ± 5.8^b^	96.9 ± 9.3^b^	94.9 ± 5.1^b^	94.3 ± 9.7^b^

^a^*p* < 0.05 was compared with group N, and ^b^*p* < 0.05 was compared with T0

### Changes of noradrenaline (NE), adrenaline (E), cortisol (COR) and blood glucose (BS)

Compared with T0 time, NE, E, Cor and BS decreased at T4 in group D, DP and P(*p* < 0.05); But there was no significant difference among NE, E, Cor, BS at T6 in group D and DP (*p* > 0.05); Compared with T0 time, Cor at T6 in group P was statistically significant (*p* < 0.05). While compared with T0 time, there were no significant difference in E, cor and BS in group N before anesthesia induction intubation (T4) (*P* > 0.05), But the values of NE, Cor, E, BS increased significantly higher at 3 min after intubation (T6) than T0 (*p* < 0.05), as shown in Table 5.

**Table 5:** Comparison of stress hormones in four groups ($\overline{x}\pm s$)

Measurements	Group	T0	T4	T6
E (ng/L)	D	198.8 ± 14.6	161.7 ± 12.1^abc^	194.3 ± 11.2^ac^
	DP	201.4 ± 16.3	129.4 ± 10.7^ab^	166.7 ± 14.7^a^
	P	193.0 ± 12.9	131.7 ± 11.7^abc^	188.7 ± 10.6^ac^
	N	194.7 ± 13.9	195.9 ± 14.6^c^	243.6 ± 16.3^bc^
NE (ng/L)	D	332.8 ± 2.9	328.5 ± 2.4^ab^	333.5 ± 3.2^ac^
	DP	331.9 ± 1.9	336.5 ± 2.1^ab^	337.5 ± 3.2^a^
	P	333.4 ± 3.3	376.2 ± 3.1^ab^	433.3 ± 2.9^ac^
	N	334.6 ± 2.1	385.9 ± 3.4^c^	441.6 ± 1.7^bc^
Cor (ng/mL)	D	239.5 ± 30.7	208.7 ± 37.6^ab^	244.7 ± 41.1^a^
	DP	240.6 ± 35.1	199.1 ± 32.1^ab^	240.1 ± 40.3^a^
	P	238.3 ± 33.6	209.8 ± 35.3^ab^	285.3 ± 44.7^b^
	N	241.6 ± 35.1	240.4 ± 31.1^c^	292.1 ± 31.3^bc^
BS (mmol/L)	D	4.78 ± 0.26	4.31 ± 0.32^ab^	4.79 ± 0.28^a^
	DP	4.75 ± 0.38	4.28 ± 0.24^ab^	4.57 ± 0.35^a^
	P	4.71 ± 0.23	4.37 ± 0.42^ab^	4.77 ± 0.38^a^
	N	4.76 ± 0.51	4.77 ± 0.62^c^	5.19 ± 0.56^bc^

^a^*p* < 0.05 was compared with group N, ^b^*p* < 0.05 was compared with T0, and ^c^*p* < 0.05 was compared with group DP

### Comparison of adverse reactions among four groups

There was no significant hypotension in the four groups. In group N, 5 cases had cough (16.67%) and 3 cases had arrhythmia (10%). Compared with group N, group D had 1 arrhythmia (3.33%) with no statistical significance (*p* > 0.05) which returned to normal level after intravenous injection 0.5 mg atropine; group D had 2 cough (6.67%) with statistical significance (*p* < 0.05); group P had 1 arrhythmia (3.33%) with statistical significance (*p* < 0.05); group DP had no no adverse reaction with statistically significant (*p* < 0.05). See Table 6 for details.

**Table 6:** Comparison of intubation stress response in the four groups (n%)

Group	Choking (%)	Vomiting(%)	Arrhythmology(%)
D	2 (6.67)	0 (0)	1 (3.33)
DP	0 (0)	0 (0)	0 (0)
P	1 (3.33)	0 (0)	1 (3.33)
N	5 (16.67)	0 (0)	3 (10.00)

## Discussion

According to the relevant medical data statistics, the incidence rate of sinusitis and nasal polyps in China has been increasing [3] in recent years. Surgery is an ideal method for the treatment of patients with sinusitis and nasal polyps. In early clinical practice, conventional sinus surgery is often used to treat the disease. However, it is found that this kind of surgery causes greater damage to the patients’ nasal mucosa, and postoperative complications such as nasal adhesion likely to occur, which is not conducive to the recovery of patients [4]. With the continuous progress of medical technology, functional endoscopic sinus surgery (FESS) has been widely used in the clinical treatment of patients with sinusitis and nasal polyps. Compared with conventional sinus surgery, endoscopic sinus surgery has the advantages of simple operation, small trauma, low postoperative complications, and is more and more trusted by the majority of patients [5].

Previous studies have shown that the incidence of preoperative anxiety in perioperative patients is generally about 60%, up to 80% [6]. When preoperative patients have emotional reactions of over tension and anxiety, it will directly affect the smooth operation and postoperative physical recovery [7, 8]. Therefore, the ideal anesthesia goal should be anxiety relief before operation, stable hemodynamics during operation with less bleeding and clear operation field, quick recovery after operation. Make the patient safe and comfortable during the whole perioperative period.

Dexmedetomidine is a highly effective and selective agonist of adrenergic α 2 receptor, which can stimulate the blue spots of brain stem in α 2 receptor in nervous system to play an analgesic role, and can also act on α 2 receptor in presynaptic membrane and postsynaptic membrane of spinal cord hind foot to produce a sedative effect similar to natural sleep [9, 10]. Compared with midazolam, it has its unique advantages: stable hemodynamics, inhibition of sympathetic activity and analgesia. It is a new sedative and analgesic drug [11–14]. In addition, it is convenient to increase the depth of anesthesia during the operation, reduce the dosage of other anesthetics, and maintain a relatively stable vital signs [15]. When it acts on the nucleus tractus solitarius of medulla oblongata, it will produce sympathetic inhibition, resulting in hypotension and bradycardia [16]. The results of this study showed that Ramsay Sedation in group P and group N was 1–2 points, BIS value was greater than 89, this suggests that group P and group N patients were in awake state; Ramsay Sedation in group D and group DP was maintained at 2–4 points, BIS value was about 70–80, and this suggests that patients of group D and group DP were in shallow sedation state, showed calm, responded accurately to language instructions, and were in a sleep state without external stimulation, but they were easily awakened by language stimulation, cooperated and communicated with medical staff, and soon entered a sleep state after the stimulation disappeared.

Previous studies have shown that the application of DEX before anesthesia can reduce or eliminate the preoperative anxiety of patients to the greatest extent, so that they can actively cooperate and successfully pass the perioperative period [17]. The results of this study are consistent with the state anxiety score SAI. Therefore, We think that the preoperative application of DEX can not only calm the patients, but also reduce their anxiety response. It also provides the relevant basis for how to reduce the incidence of preoperative anxiety and the stress induced by preoperative anxiety. Good preoperative medication not only needs to relieve patients’ anxiety, fully calm and produce amnesia, but also needs to reduce the stress of nerve reflex and stabilize hemodynamics. Therefore, how to regulate stress response reasonably should be concerned by anesthesiologists. Perfect preoperative medication can make the hemodynamics induced by anesthesia stable, and reduce the stress response caused by placing laryngoscope and tracheal intubation [18]. Parecoxib sodium is a kind of water-soluble non steroidal anti-inflammatory drugs (NSAIDs). It is a new analgesic drug. It is a second generation selective inhibitor of cyclooxygenase-2 (COX-2). It can be injected intravenously or intramuscularly and then transformed into vardixib. The research shows that the analgesic effect is accurate, it can effectively control the acute pain caused by surgical trauma, reduce the postoperative pain score, and improve the patient satisfaction rate [19]. The analgesic mechanism is that it can effectively inhibit the activity of COX-2, inhibit the activation of ERK in spinal neurons, and play an analgesic and anti-inflammatory role. Previous studies have shown that preoperative administration of parecoxib sodium can reduce the production of various inflammatory mediators and cytokines, reduce the sensitivity of nerve center and peripheral to pain, and improve the quality of pain control [20]. According to the results of this study, the intubation was successful in four groups after induction of conventional anesthesia. The SBP, DBP, HR in the control group were significantly higher than those in the control group at 3 min (T6) and 5 min (T7) after intubation. The SBP, DBP in group P and N at the time of intubation (T5) and 3 min after intubation (T6) were significantly higher than before administration (T0), while the SBP, DBP in group DP and D at the time of intubation (T5) and 3 min after intubation (T6) kept the same level as the before administration (T0). Compared with before administration (T0), HR of group D and DP decreased significantly at the time of intubation (T5), 3 min after intubation (T6) and 5 min after intubation (T7), while HR of group N increased significantly at T5, T6 and T7. HR of group P did not change significantly at each time point. We speculate that DEX can reduce the self-regulation of sinoatrial node by selectively acting on renal α 2-adrenergic receptor of myocardium, so that the body does not have the increase of heart rate after stress.

Our results showed that the NE, E, Cor and BS of group D, DP and P decreased before anesthesia induction (T4), while the NE, E, Cor, BS of group DP decreased more significantly than those of group D. And compared with before T0, the NE, E, Cor, BS of group D, DP and P remained at the same level with T6. While compared with T0, the NE, E, Cor, BS of group N had no significant difference from T4, but the values significantly increased at T6.Compared with group DP, NE and E of group D, P and N were statistically significant at T6.The concentrations of NE, E, Cor and BS are good indicators of “alert period” of stress response, and the degree of increase is positively correlated with the stimulation intensity of stress. The results showed that the control group did not inhibit the stress response of tracheal intubation well, and group DP was more effective in inhibiting the stress response of tracheal intubation.

Since the anesthesiology department of our hospital began to use DEX and parecoxib sodium, through our continuous learning and clinical practice, our research group has confirmed in the early clinical research that the intranasal drip of DEX (1–1.5μg / kg) in 45 min before gynecological laparoscopic surgery can achieve the “wake-up” sedation and anti anxiety effect for patients before surgery [21]. Pre-injection of Dex in thyroid surgery can improve the stability of hemodynamics, reduce the stress response of patients, inhibit the over secretion of PRA, ANG-II and ALD, which is conducive to the stability of circulatory system in the recovery period of patients with general anesthesia in thyroid surgery, and does not extend the recovery time [22]. We found that these two drugs not only have good “sober and sedative”, analgesic and protective effects on organs such as heart and brain, but also have many beneficial effects such as stabilizing hemodynamics, inhibiting perioperative stress response, reducing the dosage of anesthetics and analgesics, and reducing postoperative discomfort of patients, which are worthy of further discussion in clinical practice. At present, there are few reports about it in the domestic and foreign literature, lacking of the corresponding clinical application standards, norms and the best dosage suitable for Chinese constitution. Jian Lu et al. [23] evaluate the effect of parecoxib sodium pretreatment combined with dexmedetomidine on early postoperative cognitive dysfunction in elderly patients after shoulder arthroscopy. But perhaps because of the different goals of the study, all of their patients were treated with parecoxib sodium without a control group. Niyogi S et al. [24] compared the efficacy of intranasal and intravenous DEX to attenuate the stress response of laryngoscopy and endotracheal intubation.they found that there was no significant differences between intranasal and intravenous DEX. However, the long effective time of intranasal administration should also consider the absorption of nasal mucosa at FESS operation site. Given the procedure short median time of FESS and considering the efficient administration of Dex via nasal route [25–27], in order to minimize Operation occupation time, We used the intravenous route. Our results show that intravenous injection of dexmedetomidine combined with parecoxib sodium 30 min before FESS operation can not only achieve sedation and anti anxiety, but also better prevent tracheal intubation stress response. It is a safe and effective way of preoperative medication, providing a direct clinical basis and reference for anesthesiologists to implement comfortable and safe anesthesia.

## Conclusions

Intravenous injection of dexmetomide combined with parecib sodium before functional endoscopic sinus surgery can not only calm and resist anxiety, but also better prevent stress response of endotracheal intubation. It is a safe and effective preoperative drug.

## Data Availability

The datasets generated and/or analyzed during the current study will be available from the corresponding author on reasonable request.

## Abbreviations

ASA: American Society of Anesthesiologists
BMI: Body mass index
FESS: Functional endoscopic sinus surgery
DEX: Dexmedetomidine
SPO_2_: Oxygen saturation
BIS: Bispectral index
Cor: Circulating cortisol
E: Epinephrine
NE: Norepinephrine
BS: Blood sugar
